# Tumor Necrosis Factor-Alpha Antagonist Interferes With the Formation of Granulomatous Multinucleated Giant Cells: New Insights Into *Mycobacterium tuberculosis* Infection

**DOI:** 10.3389/fimmu.2019.01947

**Published:** 2019-08-14

**Authors:** Soraya Mezouar, Issa Diarra, Jean Roudier, Benoit Desnues, Jean-Louis Mege

**Affiliations:** ^1^Aix-Marseille Université, IRD, APHM, MEPHI, Marseille, France; ^2^IHU-Méditerranée Infection, Marseille, France; ^3^Department of Rheumatology, Institut du Mouvement et de l'appareil Locomoteur, APHM, Marseille, France; ^4^APHM, IHU Méditerranée Infection, UF Immunologie, Marseille, France

**Keywords:** etanercept, granuloma, *Mycobacterium tuberculosis*, multinucleated giant cells, interleukine-17, interleukin-10, tumor necrosis factor

## Abstract

More than half of tuberculosis cases in the world are due to resuscitation of dormant *Mycobacterium tuberculosis* (*Mtb*) sequestered into cell-derived structures called granulomas. It is fairly admitted that cytokines and more particularly Tumor Necrosis Factor (TNF)-α is critical in the control of *Mtb* infections and that anti-TNF-α drugs constitute one of the main risk factors for reactivation of latent *Mtb* infection. The aim of this study was to evaluate the role of etanercept, a dimeric fusion protein consisting of the extracellular ligand-binding portion of the human p75 TNF receptor linked to the Fc portion of human IgG1, in an *in vitro* model of human tuberculous granuloma. We showed that etanercept slightly delayed the formation of granuloma and reduced the generation of multinuclear giant cells (MGCs). In addition, etanercept exacerbated the expression of M1 polarization genes but also induced interleukin (IL)-10 release. In addition, our results indicated that etanercept inhibited cell fusion in an IL-10-dependent manner. Moreover, adalimumab, a human monoclonal anti-TNF-α IgG1 inhibited MGC formation in granuloma, without altering IL-10 secretion and induced macrophage apoptosis. Taken together, our data provides new insights into the role of TNF-α blockers in MGCs formation and the impact of such immunomodulatory drugs on tuberculous granuloma maturation.

## Introduction

Tuberculosis is an infectious disease caused by *Mycobacterium tuberculosis* (*Mtb*), which remains a major threat in terms of mortality and morbidity. While nearly one fourth of the global population is latently infected by *Mtb* (1.7 billion individuals), only 5–10% of infected people develop active tuberculosis (1). Most exposed individuals remain asymptomatic and are referred as latent tuberculosis individuals (2). Reactivation of tuberculosis depends on high-risk factors such as poverty, promiscuity, diabetes, malnutrition, immunodeficiency, or human immunodeficiency virus (HIV) infection (3). Protective immunity against *Mtb* requires efficient innate and adaptive immunity. Infection of macrophages and dendritic cells by *Mtb* leads to T cell activation and cytokine production (4, 5), among which interleukin (IL)-12 and interferon (IFN)-γ have been shown essential for the protection against *Mtb* as revealed by murine models and human immune deficiencies (3). In addition, among cytokines secreted by *Mtb*-activated immune cells and infected individuals, tumor necrosis factor (TNF)-α has been considered as necessary for bacterial killing (6).

The hallmark of the immune response to *Mtb* is the formation of an organized cellular structure called granuloma to control the infection. In the early stage, granulomas exhibit a core of infected macrophages enclosed by foamy macrophages and surrounded by lymphocytes. Mature granulomas develop a fibrous capsid isolating macrophage core and reducing vascularization, thereby restraining *Mtb* dissemination, without overt symptoms in patients (7). Disease progression from latent to active tuberculosis is associated with a defect of the host immune response to control the infection. Several high-risk factors reviewed in Ai et al. (8) have been shown to significantly increase latent tuberculosis rate and includes HIV infection (9), organ transplantation with use of immunosuppressive drugs (10), silicosis (11), contact with active tuberculosis patients (12), TNF-α blockers (13), and hemodialysis in patients with chronic renal failure (14). Latent tuberculosis reactivation involves caseous necrosis of macrophages in mature granulomas; caseous center then liquefies and allows the release infectious *Mtb* in the airways (15–17).

Among the diversity of immune effectors involved in granuloma formation, IFN*-*γ and TNF-α are considered as positive regulators whereas IL-10 is a negative regulator (18). Center to the mycobacterial granuloma formation is the remodeling of macrophages. Indeed, granuloma foamy macrophages represent 10–20% of the total macrophages and are characterized by intracytosolic accumulation of neutral lipids forming lipid bodies, also known as lipid droplets or lipid vacuoles (17, 19–21). In addition, macrophages can fuse to form multinucleated giant cells (MGCs) (22). Although the mechanisms leading to MGC formation are poorly understood, cytokines such as IFN*-*γ, IL-4, IL-10, and IL-17 have been involved (5, 23–26). However, the limited clinical material availability may explain why functional studies remain scarce. In addition, animal models of granulomas provide divergent results. Indeed, while murine models confirmed the protective role of granuloma, zebrafish model of *M. marinum* infection reappraised protective role for granulomas (27). Thus, *in vitro* models of granulomas have been developed by co-culturing peripheral blood mononuclear cells (PBMCs) and Sepharose beads coated with bacterial extracts from *Mtb* or *M. bovis* (16, 28, 29). Using this approach, we previously showed that monocytes migrate to the beads, maturate into macrophages which then polarize and fuse to form MGCs under the influence of lymphocytes (30, 31). In addition, we also showed that defective granuloma formation was associated with low TNF-α expression and monocytopenia in septic patients (32).

Several studies have highlighted the role of TNF-α in the formation and the stability of granuloma (33, 34). Other data have shown that TNF*-*α blockade, by the use of TNF-deficient mice or anti-TNF*-*α drugs, induced delayed formation of granuloma, necrosis, disorganization, or disintegration of granuloma structures (35, 36). Kapoor et al. showed that treatment of *in vitro Mtb* granuloma with anti-TNF-α was associated with the reactivation of latent *Mtb* (37). These observations suggest that anti-TNF-α interfere with granuloma formation and/or stability. Interestingly, clinical observations revealed that the risk of tuberculosis reactivation is associated with anti-TNF-α treatment but also depends on the type of anti-TNF-α agent. Indeed, monoclonal antibodies, such as infliximab or adalimumab are associated with a 5–10-fold increased risk of reactivation of tuberculosis, while etanercept, which consist of a fusion protein between two extracellular domains of the human TNF receptor 2 and the Fc fragment of human IgG1, is associated with no or only few cases of tuberculosis reactivation (13, 38–41). Interestingly, experimental investigations suggested that etanercept prevent complement activation and cell death but also preserve granuloma formation whereas anti-TNF-α antibodies did not (42). In addition, it has been showed that etanercept treatment impact on the remodeling process involved in the formation and the maintenance of granuloma in a *Mtb* infection using rabbit model (43).

Thus, the aim of this study was to clarify the effect of etanercept on granuloma formation. Using an *in vitro* model of granuloma formation, we showed that etanercept treatment did not alter granuloma formation. Interestingly, we report here that etanercept treatment affects granulomatous macrophage population and polarization and inhibits MGC formation in an IL-10-dependent mechanism.

## Materials and Methods

### Bacteria Culture and Preparation of Bacterial Extracts

*Mycobacterium tuberculosis* (H37Rv) was cultivated in Middlebrook 7H10 agar medium supplemented with 10% of Oleic acid-albumin-dextrose-catalase (OADC) (Beckman Dickinson). Bacteria (10^9^ per assay) were sonicated in a coupling buffer (NaHCO_3_ 0.1 M pH 8.3 with NaCl 0.5 M) for seconds at 70% amplitude five times (Vibra Cell 75185) and protein concentration was determined by Nanodrop as previously described (30).

### Isolation of PBMCs and Granuloma Formation

Human blood was obtained from leukopack left over from voluntary whole blood donations after informed consent of the donors according the convention *n*°7828 established between our laboratory and the “Etablissement Français du Sang” (Marseille, France). PBMCs were recovered using density gradient centrifugation as previously described (16, 28, 44, 45). Isolated PBMCs (2 ×10^5^ cells/well) were suspended in RPMI 1,640 medium supplemented with 10% fetal calf serum (FCS, Life Technologies), 100 IU/ml penicillin and 50 μg/ml streptomycin (Life Technologies) and incubated with activated 4B Sepharose beads that were previously coated with [*Mtb*] extracts (0.5 mg of proteins) at 37°C with 5% CO_2_. The kinetics of granuloma formation was evaluated using inverted microscope after 3, 6, and 9 days of culture in the presence or absence of etanercept, a humanized soluble recombinant TNF receptor fusion protein. In some experiments, we used adalimumab, a human IgG1 monoclonal antibody directed against TNF-α. According to the therapeutic range of residual serum concentration of anti-TNF-α in treated patients, we used 10 μg/ml of anti-TNF-α drugs in our experiments, as previously described (46–48). Concentration of anti-TNF-α drugs is maintained at concentration 10 μg/ml during the kinetics of granuloma formation.

### RNA Extraction and Real-Time Quantitative RT-PCR (qRT-PCR)

Individual granulomas were manually collected, and granuloma cells were dissociated by incubation with phosphate-buffered saline (PBS, Life Technologies) buffer containing 2 mM EDTA (Invitrogen). Total RNA from granuloma cells was extracted and treated with DNase using RNeasy® Mini Kit (Qiagen). Reverse transcription was performed as previously described (49). Quantitative PCR was carried out using Light Cycler Fast Start DNA master SYBR Green I kit (Roche) and the primers listed in Table 1 (31). Real-time PCR was performed as follows: initial denaturation at 95°C for 10 min, followed by 39 cycles of denaturation at 95°C for 15 s and an annealing/extending step at 60°C for 1 min. The results were normalized with the housekeeping gene β-actin. Transcript relative quantity (RQ) and the fold change (FC) of target genes relative to β-actin were calculated using the formula RQ = 2^−ΔCt^ and FC = 2^−ΔΔCt^, respectively.

**Table 1:** Primer sequences for qRT-PCR.

**Gene symbol**	**Forward primer sequence**	**Reverse primer sequence**
β-actin	ggaaatcgtgcgtgacatta	aggaggaaggctggaagag
*IL15RA*	atcttccgtccctcatcctaac	ctcagcatctctcccaccttt
*TNFSF10*	gaaaataatccccacacacgctac	gtcactctctccaccctcaca
*SLC4A7*	ccctcaaaacagtcctccttct	tttcctcattcttctgctcctc
*CLECM4*	ggcatttctggtagagttcaca	atactttctgactgggcagga
*HESX1*	gctcggggaaaacaaacc	ttcttctggcattgggtga
*CXCL9*	acacttgcggatattctggact	gggagatggtgtgtaattgat
*IL15*	agaatgtgaggaactggaggaa	tgtctaagcagcagagtgatgt
*TNF*	catctatctgggaggggtcttc	aggagggggtaataaagggatt
*CCL13*	gagcagagaggcaaagaaaca	atgtgaagcagcaagtagatgg
*FN1*	acacctggagcaagaaggataa	ccacagagtagaccacaccagt
*HRH1*	acttggaggtggtatgtgctg	ctcagggcttgcttcttgtagt
*ALOX15*	aacttccaccaggcttctctc	gggggctgaaataaccaaag
*CTSC*	gaggttgtgtcttgtagccagt	cccctttttgtagtggaggaag
*IL2RA*	gagacttcctgcctcgtcacaag	gatcagcaggaaaacacagcc
*CCL23*	catcttcctacaccccacgaa	cattctcacgcaaacctgaact
*IDO1*	tcatctcacagaccacaagtca	caaaataggaggcagttccaagt
*IL17*	gaaacctcccaaaatacaag	taaagttcgttctgccccatc

### Immunophenotyping and Immunofluorescence Analysis

Dissociated granuloma cells were analyzed by flow cytometry to identify macrophages and lymphocyte populations with appropriate antibodies and isotype controls listed in Table 2. For apoptosis assay, granuloma cells were labeled with an anti-CD64 antibody (Beckman coulter) following by Annexin-V/7AAD staining according to the manufacturer instructions (BioLegend). Flow cytometry analysis were performed on a BD Canto II and data were analyzed using Flow Jo software.

**Table 2:** List of fluorescent reagents (mouse IgG1 antibodies).

**Antibody**	**Clone**	**Fluorochrome**	**Manufacturer**
CD68	KP1	FITC	Dako
CD163	GHI/61	PE	Becton Dickinson
CD56	N901	APC	Beckman Coulter
CD8	SK1	PerCP/Cy5.5	Bio Legend
CD3	SK7	APC-H7	BD Pharmingen
CD4	13B8.2	FITC	Beckman Coulter
CD20	B9E9	PC7	Beckman Coulter
CD127	HIL-7R-M21	PE	BD Pharmingen
IL-17	BL128	Pacific Blue	BD bioscience

May-Grünwald-Giemsa (MGG) staining was used to identify mononuclear and multinuclear giant cells on dissociated granuloma cells. The percentage of mononuclear and multinuclear giant cells was quantified using an inverted microscope after 9 days of culture in the presence or absence of anti-TNF-α agent (5 different fields were analyzed for each condition).

For immunofluorescence, cells were fixed in 4% paraformaldehyde in PBS for 20 min and stained with Phalloidin and 4′,6-diamidino-2-phenylindole (DAPI) (both from Thermo Fisher Scientific) to label filamentous actin and DNA, respectively. Pictures were observed on LSM 800 Airyscan confocal microscope (Zeiss) with a 63X oil objective.

### Cytokine Measurement

IL-10, IL-6, TNF-α, IL-17, and IFN-γ were quantified in cell supernatants using specific ELISA kits and processed according to the manufacturer's instructions (eBioscience, Clinisciences and R&D systems). Supernatants were collected at day 9 after cell stimulation.

### Cell Fusion Assay

CD14^+^ monocytes were selected from PBMCs using CD14-microbeads (Miltenyi) and cells (5 ×10^5^) were stimulated with IFN-γ (100 U/ml, Biolegend) and concanavalin (ConA, Sigma-Aldrich, at 5 μg/ml) as previously described (50, 51) in the presence or not of etanercept (10 μg/ml), isotype control, IL-17 [10 ng/ml, PreproTech, (52)], IL-10 [50 ng/ml, R&D systems, (26)] or TNF-α (50 ng/ml, Euromedex) and incubated for 9 days.

### Statistical Analysis

All experiments have been repeated at least 3 times. Data were analyzed using GraphPad Prism 5 (GraphPad Software, Inc.) and Mann-Whitney *U* test. Results were presented as mean ± standard error of the mean (SEM) and were considered significant at *P* ≤ 0.05.

## Results

### Etanercept Does Not Affect Tuberculous Granuloma Formation

**Figure 1:**
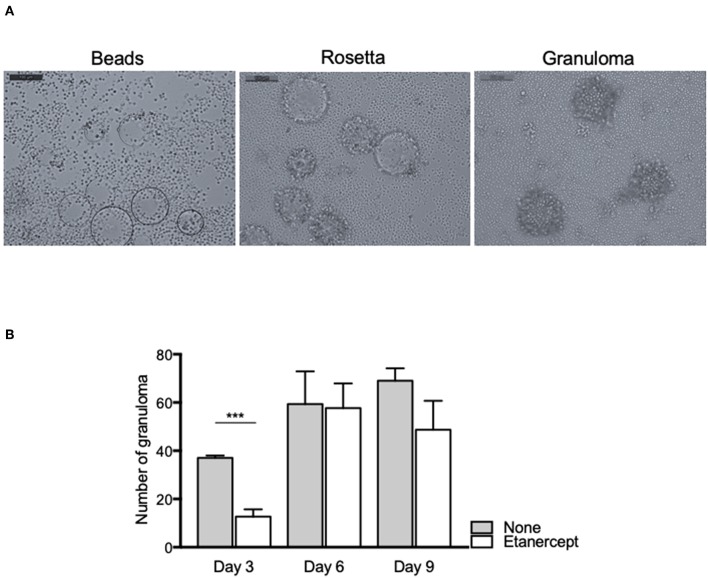
Etanercept delays the formation of tuberculous granulomas. Isolated PBMCs from healthy donors were incubated with Sepharose beads coated with *Mtb* extracts for different periods of time in the presence or not of etanercept. **(A)** Representative pictures of beads, rosetta and granuloma are shown. **(B)** The number of granulomas was counted after 3, 6, and 9 days and shown as mean ± SEM (*n* = 10) ***P < 0.001.

In order to investigate the effect of etanercept in *Mtb*-induced granuloma formation, we used an *in vitro* model of granuloma with Sepharose beads as previously described (16, 31). As depicted in Figure 1A, the use of beads coated with *Mtb* extract lead to a recruitment of PBMCs around the beads first and initiating the formation of rosetta and then of granuloma, defined as bead fully covered with cells. PBMCs from healthy donors were incubated with *Mtb* extract-coated beads in the presence or not of etanercept and granuloma formation was followed over time. In untreated conditions, nearly 40% of the beads display a granulomatous structure after 3 days and this percentage steadily increased to reach 70% after 9 days. In contrast, when cells were treated with etanercept, the formation of granuloma was significantly decreased (21.5%) at day 3 as compared with untreated cells (Figure 1B). However, after 6 and 9 days, the percentage of granuloma was similar in the presence or absence of etanercept (Figure 1B and Supplemental Figure 1). Hence, these data suggest that etanercept does not affect but delays *in vitro* granuloma formation.

### Etanercept Affects the Formation of Multinucleated Giant Cells

**Figure 2:**
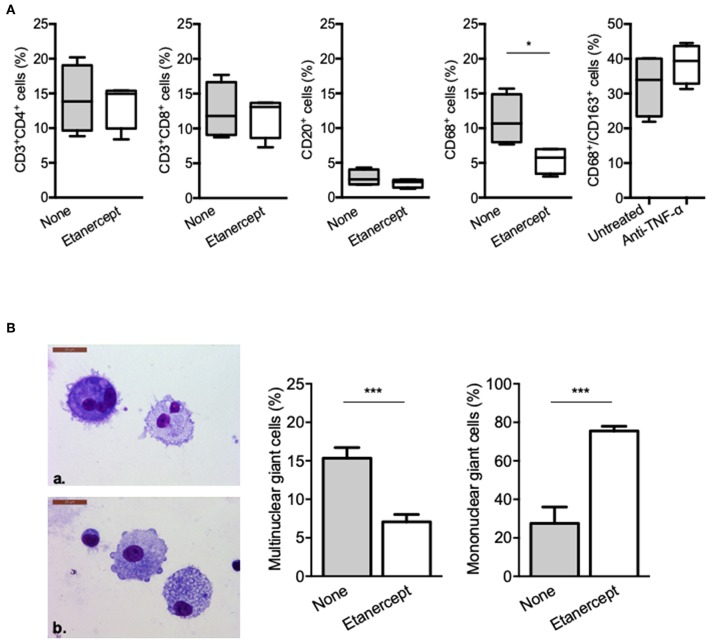
Etanercept affects granuloma-associated macrophage populations and MGCs. Isolated granuloma cells cultured in the presence or not of etanercept for 9 days were characterized using flow cytometry. **(A)** Graphical plots of the percentage of CD3^+^/CD4^+^, CD3^+^/CD8^+^, and CD20^+^ lymphocytes and CD68^+^ and CD68^+^CD163^+^ macrophages population (*n* = 4) *P < 0.05. **(B)** Isolated granuloma cells cultured in the presence or not of etanercept for 9 days were characterized by May-Grünwald-Giemsa staining. Representative image of multinuclear (upper panel) and mononuclear (lower panel) cells are shown, quantified and represented as the mean percentage ± SEM (*n* = 10) ***P < 0.001.

We next wondered whether granuloma composition was affected. PBMC and *Mtb* extract-coated beads were co-cultured with or without etanercept and the composition of the cell aggregates was assessed after 9 days by flow cytometry for CD4^+^ and CD8^+^ T cells, B cells and macrophages or May-Grünwald-Giemsa staining for multinucleated giant cells (MGCs). In untreated conditions, granulomas were composed of 14.19 ± 2.43% of CD4^+^ T cells, 12.51 ± 2% of CD8^+^ T cells, 2.84 ± 0.57% of B cells, and 11.18 ± 1.81% of CD68^+^ macrophages (Figure 2A). Treatment with etanercept did not affect lymphocytic composition of cell aggregates. In contrast, it significantly reduced the proportion of CD68^+^ macrophages without affecting the expression of CD163 (Figure 2A). Finally, May-Grünwald-Giemsa staining revealed that in untreated co-cultures, 17 ± 2% of the granuloma cells were MGCs containing 2–3 nuclei. Interestingly, in the presence of etanercept, multinuclear GC proportion was significantly reduced while the number of mononuclear giant cells was increased (Figure 2B). Taken together, these results suggest that etanercept affects the formation of MGCs in tuberculous granulomas.

### Etanercept Affects M1/M2 Polarization of Granuloma Cells

**Figure 3:**
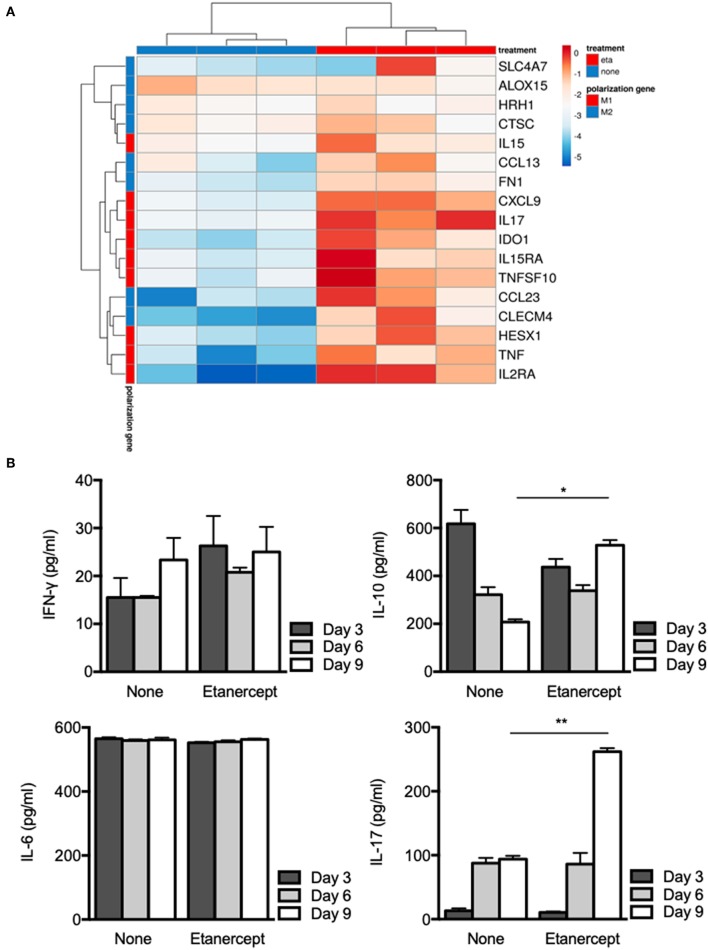
Etanercept affects macrophage polarization. Isolated PBMCs from healthy donors were incubated with *Mtb* extract-coated beads for different periods of time in the presence or not of etanercept **(A)** The expression of macrophage polarization genes was investigated by quantitative RT-PCR normalized to the actin endogenous control and displayed as heat-map. **(B)** IFN-γ, IL-6, IL-10, and IL-17 cytokines were quantified by ELISA at day 3, 6, and 9 in supernatants from *in vitro* tuberculous granulomas treated or not with etanercept (*n* = 3) *P < 0.05, **P < 0.01.

We previously reported that BCG- and *Coxiella burnetii*-induced granulomas were characterized by expression of genes related to macrophage polarization (31). Hence, we next wondered if etanercept treatment affected the transcriptional profile of granuloma cells. PBMC from 3 donors were cultured in the presence of *Mtb* extract-coated beads with or without etanercept and macrophage polarization was evaluated after 9 days by qRT-PCR targeting M1 and M2 genes. Analysis of gene expression by hierarchical clustering clearly showed that etanercept modulated gene expression of granuloma cells since untreated and etanercept-treated cultures were localized on separated branches (Figure 3A). However, further clustering of transcripts in response to etanercept highlighted 2 main clusters (Figure 3A). The first cluster encompassed genes for which etanercept had no or discrete effect on gene expression and included the M2 genes *SLC4A7, ALOX15, HRH1, CTSC*, and the M1 gene *IL15*. In contrast, in the second cluster were found genes that were highly induced by etanercept, including the M1-associated genes *CXCL9, IL17, HESX1, IDO1, IL15RA, TNFSF10, IL2RA*, and *TNF* as well as some M2-related genes such as *CCL13, FN1, CCL23*, and *CLECM4* (Figure 3A).

Finally, we investigated cytokine release by granuloma cells treated or not with etanercept. Supernatants were collected after 3, 6, and 9 days of co-cultures and were assessed for the presence of IFN-γ, IL-6, IL-10, and IL-17. In untreated cultures, we found that IFN-γ and IL-6 levels were not really affected during the 9 days of culture, while IL-17 was increased between day 3 and day 6 and IL-10 gradually decreased from day 3 to day 9 (Figure 3B). Etanercept treatment did not alter IFN-γ and IL-6 release by granuloma cells as compared to untreated conditions (Figure 3B). However, at day 9, IL-10 was significantly increased in granuloma cells treated by etanercept as compared to untreated cells. Similarly, IL-17 levels were significantly increased confirming transcript measurements (Figure 3B). Altogether, these results suggest that inhibition of TNF-α alters macrophage polarization and cytokine release in *Mtb*-induced granulomas.

### Etanercept Inhibits the Formation of Cell-Cell Fusion-Induced MGCs

**Figure 4:**
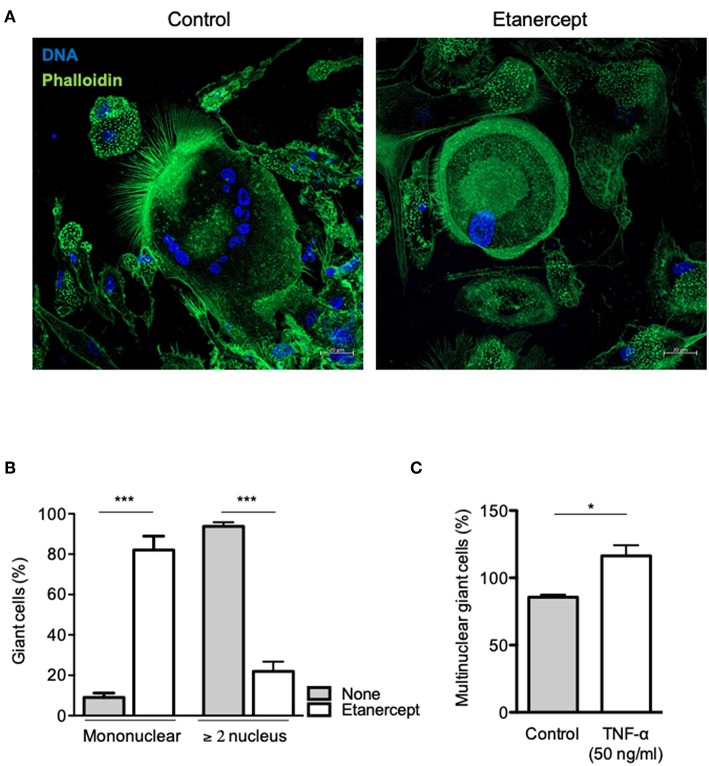
Etanercept inhibits cell fusion. Adherent CD14^+^ cells from healthy donors (*n* = 5) were stimulated with IFN-γ and ConA in the presence or not of etanercept and cell-cell fusion was measured by the appearance multinucleated giant cells (≥2 nucleus). **(A)** Representative pictures of multinucleated cells stained with phalloidin (green) and DAPI (blue). **(B)** Mononuclear and multinuclear (≥ 2 nucleus) giant cells were quantified after 9 days and the mean percentage ± SEM is shown (*n* = 5) ***P < 0.001. **(C)** Formation of multinucleated cells induced by IFN-γ and ConA was also quantified in the presence of 50 ng/ml TNF-α (*n* = 5) *P < 0.05.

To further clarify the role of TNF-α in MGC formation, we performed a cell-cell fusion assay mediated by ConA and IFN-γ stimulation. Adherent CD14^+^ cells from healthy donors were treated with IFN-γ and ConA and cell-cell fusion was measured by the appearance of MGCs (≥2 nuclei). As depicted in Figure 4A, MGCs (2–15 nuclei) were found in untreated cultures at day 9 after ConA/IFN-γ stimulation. In contrast, treatment with etanercept decreased the number of MGCs and was associated with the appearance of large mononuclear cells (>60 μm, Figure 4A). After quantification, we showed that upon etanercept treatment, the number of these large mononuclear cells increased from 10 to 80% while that of MGCs decreased from 90 to 20% as compared with untreated cells (Figure 4B). Hence, these results suggested that etanercept interferes with the formation of MGCs and that TNF is required for the development of MGCs. To confirm the role of TNF-α in MGCs formation we cultured CD14^+^ cells with ConA and IFN-γ in the presence of TNF-α. As shown in Figure 4C, the addition of TNF-α significantly increased the percentage of MGCs as compared with cells cultured with ConA and IFN-γ alone. Taken together, these data showed that TNF-α is required for the formation of MGCs and that inhibition of TNF-α by etanercept favors the development of large mononuclear cells.

### IL-10 Inhibits the Formation of MGCs

**Figure 5:**
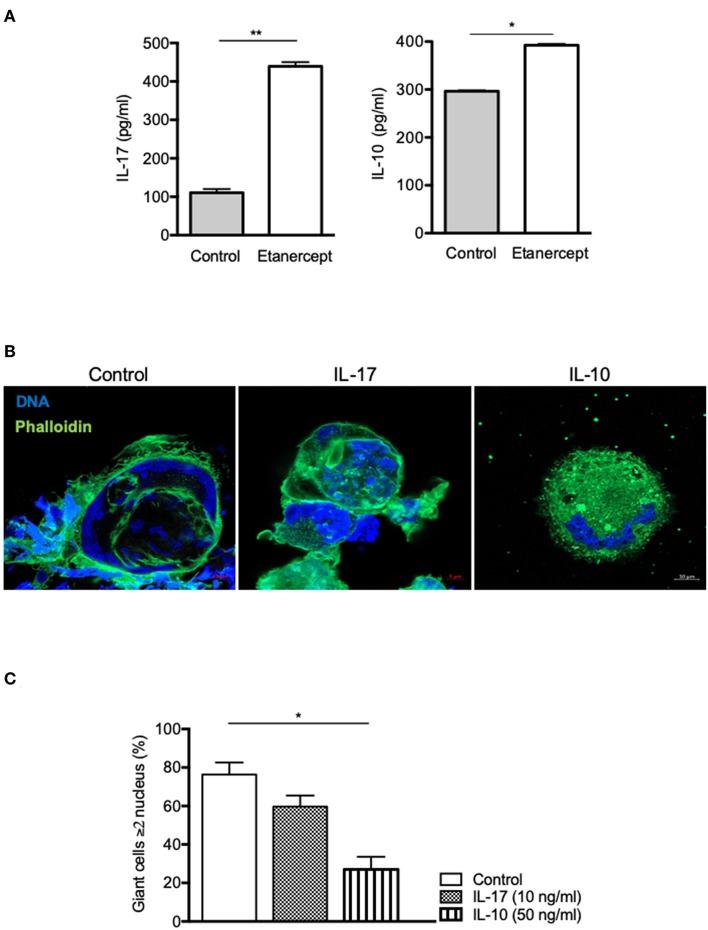
IL-10, not IL-17, inhibits cell fusion. **(A)** Adherent CD14^+^ cells from healthy donors (*n* = 5) were stimulated by IFN-γ and ConA in the presence or not of etanercept and IL-17 and IL-10 concentration were evaluated by ELISA in the supernatants after 9 days *P < 0.05, **P < 0.01. **(B,C)** Adherent CD14^+^ cells from healthy donors were stimulated by IFN-γ and ConA in the presence or not of IL-17 (10 ng/ml) or IL-10 (50 ng/ml) for 9 days. **(B)** Representative pictures of MGCs stained with phalloidin (green) and DAPI (blue) are shown. **(C)** Formation of MGCs was also quantified and the mean percentage ± SEM is shown (*n* = 4) *P < 0.05.

As IL-17 and IL-10 expression and secretion were strongly increased in *Mtb*-induced granuloma upon etanercept, we wondered if it was also modulated in cell-cell fusion assay. Hence, we measured IL-17 and IL-10 in supernatants from ConA/IFN-γ-treated PBMCs and found that anti-TNF-α treatment lead to a 4-fold and a 1.3-fold increase of IL-17 and IL-10 release, respectively as compared with untreated cells (Figure 5A). To further understand their role in MGC formation, we performed cell-cell fusion assay in the presence of IL-17 or IL-10. As shown in Figures 5B,C, we found that IL-10, but not IL-17 affected the development of MGCs.

### Etanercept and Adalimumab Inhibit Differently MGC Formation

**Figure 6:**
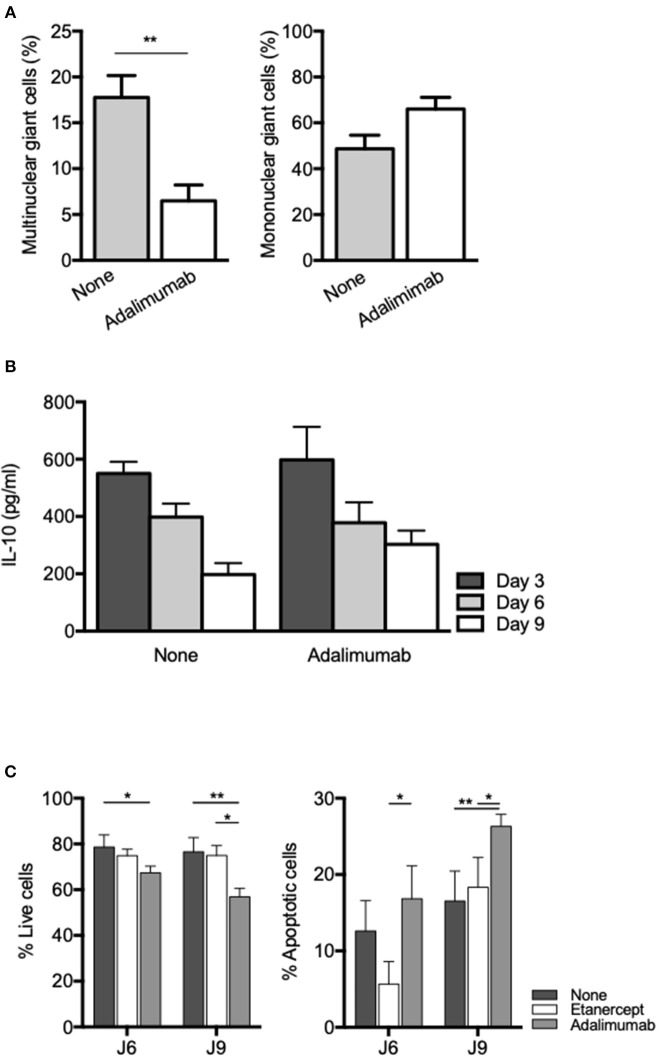
Adalimumab inhibits MGC formation and induces macrophage apoptosis in granuloma. Isolated PBMCs from healthy donors were incubated with Sepharose beads coated with *Mtb* extracts in the presence or not of adalimumab. **(A)** After 9 days, isolated granuloma cells were characterized by May-Grünwald-Giemsa staining and mononuclear and multinuclear giant cells were quantified (*n* = 3). **P < 0.05. **(B)** IL-10 levels were quantified by ELISA at day 3, 6, and 9 in supernatants from *in vitro* tuberculous granulomas treated or not with adalimumab. **(C)** Isolated PBMCs from healthy donors were incubated with Sepharose beads coated with *Mtb* extracts in the presence or not of adalimumab or etanercept. Macrophage apoptosis was assessed by flow cytometry after annexin V/7-AAD staining on CD64-gated cells. Results are expressed as mean percentage ± SEM (*n* = 3) *P < 0.05, **P < 0.01.

TNF blockade using rabbit polyclonal antibodies or human monoclonal antibodies (adalimumab) has also been shown to inhibit formation of MGC in ConA-induced cell-cell fusion experiments (53, 54). In order to see if inhibition of MGC formation in tuberculous granuloma was restricted to etanercept or a common feature of TNF inhibition, we co-cultured PBMC and *Mtb*-extract coated beads in the presence of adalimumab, a fully human IgG1 monoclonal antibody. We found that adalimumab also lowered the percentage of Multinuclear GC (Figure 6A) to levels similar to those obtained when cells were treated with etanercept (Figure 2B). As IL-10 was associated with reduced MGC formation in etanercept-treated co-cultures (Figure 3B), we next wondered if adalimumab treatment affected IL-10 expression. Supernatants were collected after 3, 6, and 9 days and assessed for IL-10 by ELISA. To our surprise, IL-10 levels were not increased in adalimumab-treated co-cultures and were similar to those measured in untreated conditions (Figure 6B), suggesting that adalimumab- and etanercept-mediated inhibition of MGC formation involves different pathways. Finally, we asked whether the decrease of multinuclear GC in anti-TNF-α-treated co-cultures was related to increased cell death of macrophages. As shown in Figure 6C, etanercept did not induce macrophage apoptosis while adalimumab treatment resulted in a significant induction of macrophage apoptosis at day 6 and 9. Altogether together, these results suggest that etanercept and adalimumab affect the formation of MGCs in tuberculous granulomas by different mechanisms, etanercept through IL-10-mediated inhibition and adalimumab through induction of macrophage apoptosis.

## Discussion

Granuloma formation is the key response of immune cells against *Mtb* infection. In this study, we aimed at characterizing the effects of the anti-TNF-α drug etanercept on the granulomatous response by analyzing granuloma formation and composition *in vitro*. Indeed, several clinical studies have shown that the use of TNF-α antagonist was associated with an increased risk of reactivation of latent tuberculosis (13). In addition, it has been suggested that reactivation is more common in patients treated with monoclonal antibodies such as infliximab, adalimumab, or golimumab than in patients treated with soluble TNF receptors such as etanercept (13). These differences have been attributed to their relative abilities to block soluble or membrane-associated TNF-α, lymphotoxin (LT)-α, or differential induction of cell death (55). However, their role and more precisely the role of etanercept in granuloma formation and composition has not been investigated.

Our results revealed that etanercept delayed the kinetic of granuloma formation at day 3 but did not alter granuloma numbers after 6 and 9 days. Same observations were made by Flynn et al. in mice after TNF-α neutralization (6, 56). Although granuloma formation was delayed at 7 days of *Mtb* infection, similar numbers were observed after 14 days between mice treated by anti-TNF-α and IgG control. However, in anti-TNF-α-treated mice, granulomas appeared less well organized and contained less epithelioid cells (56). Similarly, in a rabbit model of active pulmonary tuberculosis, etanercept exacerbated lung pathology despite the presence of intact granulomatous structures (43). Hence, TNF-α may not be involved in the formation and maintenance of granuloma *per se*, but rather implicated in tissue remodeling and control of *Mtb* growth in the lung since etanercept treatment of rabbits was associated with upregulation of genes of the inflammatory response (43). We previously showed that *C. burnetii*- and BCG-induced granuloma formation was associated with a pro-inflammatory transcriptional response (31). In line with these data, we found that etanercept treatment exacerbated macrophage polarization toward a M1 profile.

Other studies investigating the role of anti-TNF-α in granuloma formation/maintenance showed that TNF-α blockers also impact the response to infection by interfering with phagosome maturation and/or by modulating apoptosis and cell death of immune cells (57). Upon etanercept, granuloma cell composition was not obviously affected, except for macrophages and MGCs, which were significantly decreased. MGCs are suspected to be involved in the limitation of tuberculosis infection although it has been showed that they have lost their ability to engulf bacteria (29). To date and to the best of our knowledge, there are no clinical evidences associating MGCs reduction and tuberculosis reactivation upon treatment with TNF-α blockers. We also observed a decrease of MGCs upon adalimumab treatment. Reduction of macrophage populations at the synovial level has previously been observed in rheumatoid arthritis patients treated with TNF-α blockers and has been associated with increased macrophage-specific apoptosis as compared with untreated patients (58). However, we did not observe increased macrophage apoptosis in the presence of etanercept, while macrophage apoptosis was significantly increased in the presence of adalimumab. This difference probably results from the different structures of etanercept and adalimumab. As stated above, etanercept is a fusion protein consisting of two extracellular TNFR2 domains covalently linked to an Fc domain of human IgG1 while adalimumab is a monoclonal IgG1 anti-TNF antibody (59). Specifically, adalimumab, but not etanercept has been shown to directly promote apoptosis through reverse signaling induced by transmembrane TNF binding (60). Hence, it is probable that the reduction of MGCs in adalimumab-treated co-cultures results from macrophage apoptosis.

In this study, we found that etanercept affected the cytokine balance in *Mtb*-induced granuloma. Indeed, while IFN-γ and IL-6 levels were similar in the supernatants of day 9 granuloma cultures treated or not with etanercept, IL-10 and IL-17 were significantly increased in the presence of etanercept. This effect was specific to etanercept since adalimumab did not affect IL-10 levels as compared with untreated granuloma cultures. During mycobacterial infection, TNF-α and IL-10 play opposite roles: TNF-α improves granuloma formation and maturation whereas IL-10 neutralizes these effects (61, 62). IL-10 is known as anti-inflammatory cytokine involved in the inhibition of macrophages activation, the down-regulation of Th1 responses and antigen presentation. In granuloma formation, IL-10 acts as a negative regulator of the immune response (5) and high levels of IL-10 are strongly associated with a disorganization of granuloma structure (63). Additionally, IL-10 can suppress the immune response against *Mtb* to promote the persistence and survival of pathogen (64). Interestingly, we found that etanercept inhibited monocyte fusion in a ConA/IFN-γ-mediated cell fusion assay and increased IL-10 release in the supernatants. In addition, we showed that exogenous IL-10 inhibited cell fusion of ConA/IFN-γ-stimulated monocytes, suggesting that blockade of cell fusion by etanercept is mediated by IL-10. This hypothesis is confirmed by the fact that IL-10 has previously been shown to modulate *in vitro* MGC formation and that the effect of IL-10 was reversed by addition of anti-IL-10 (26). It was previously shown that anti-TNF-α antibodies inhibited monocyte fusion induced by ConA/IFN-γ while antibodies targeting IL-1β, IL-6, or IL-1α had no effect (54). These results were further confirmed by Maltesen et al. who showed that ConA/IFN-γ-mediated monocyte fusion was inhibited by adalimumab and that exogenous TNF-α reversed methylprednisolone-mediated inhibition of monocyte fusion (53, 54). However, none of these studies have assess the expression of IL-10 after anti-TNF-α antibody treatment and whether the involvement of IL-10 in MGC formation is specific for etanercept remains to be elucidated.

Finally, we found that etanercept increased IL-17 expression and secretion in *Mtb*-induced granuloma cultures. Anti-TNF-α agents have been shown to induce IL-17 expression in CD4 T cell from patients with juvenile idiopathic arthritis or rheumatoid arthritis (65, 66). At the molecular level, anti-TNF-α treatment results in inhibition of the anti-inflammatory molecule TNFAIP3/A20 which activate the p38 MAPK and PKC to drive IL-17 expression (67). In line with this result, we found that etanercept also increased IL-17 levels in supernatants from ConA/IFN-γ-stimulated monocytes. However, exogenous addition of IL-17 during cell-cell fusion assay did not affect the fusion rate induced by ConA/IFN-γ, suggesting that the decrease of MGCs in etanercept-treated *Mtb*-induced granuloma is not related to increased IL-17. However, IL-17 has been involved in granuloma formation in infectious and non-infectious granulomatous diseases (68–70). During mycobacterial infections, the role of IL-17 is not fully understood. Infection of IL-17-deficient mice with BCG resulted in immature granulomas characterized by impaired cellular accumulation and organization (70). However, the implication of IL-17 seems to depend on the virulence since for less virulent *Mtb* clinical isolates, the IL-17 pathway appeared dispensable for protective immunity while infection with an hypervirulent strain required IL-17 for early protective immunity (71). IL-17 expression in granuloma was considered as a T cell cytokine produced by Th17 lymphocytes but also by myeloid cells including dendritic cells, macrophages, MGCs and neutrophils (24, 72). γδ T cells also produce IL-17 in murine *Mycobacterium*-induced granuloma (70). Identification of the source(s) of IL-17 in *Mtb*-induced granulomas in the presence or not of etanercept would require further investigations.

Altogether, we showed here that etanercept slightly delays granuloma formation, exacerbates the M1 polarization program and reduces the formation of MGCs. We provide evidences that the anti-TNF-α mediated-decrease of MGC in granuloma may involve an IL-10-dependent defect of cell fusion in the case of etanercept and may result from macrophage apoptosis induction in the case of adalimumab. Further studies are needed to identify the exact mechanisms involving anti-TNF-α drugs in MGCs formation and their role in tuberculous granuloma.

## Data Availability

The raw data supporting the conclusions of this manuscript will be made available by the authors, without undue reservation, to any qualified researcher.

## Author Contributions

SM, BD, and J-LM conceived and designed the experiments. SM and ID performed experiments and analyzed the data. JR provided equipment for experimentation. SM, BD, and J-LM wrote the paper.

### Conflict of Interest Statement

The authors declare that the research was conducted in the absence of any commercial or financial relationships that could be construed as a potential conflict of interest.

## Supplementary Material

Supplemental Figure 1 The formation of tuberculous granuloma is not affected by etanercept. Isolated PBMCs from healthy donors were incubated with Sepharose beads coated with *Mtb* extracts for different periods of time in the presence or not of etanercept. Representative pictures of co-cultures at day 3, 6, and 9 are shown.
