# Supplementary figures and images for: Tumor Necrosis Factor-Alpha Antagonist Interferes With the Formation of Granulomatous Multinucleated Giant Cells: New Insights Into Mycobacterium tuberculosis Infection
Source: Front Immunol. 2019 Aug 14;10:1947. doi: 10.3389/fimmu.2019.01947 (PMC6702871; doi:10.3389/fimmu.2019.01947)

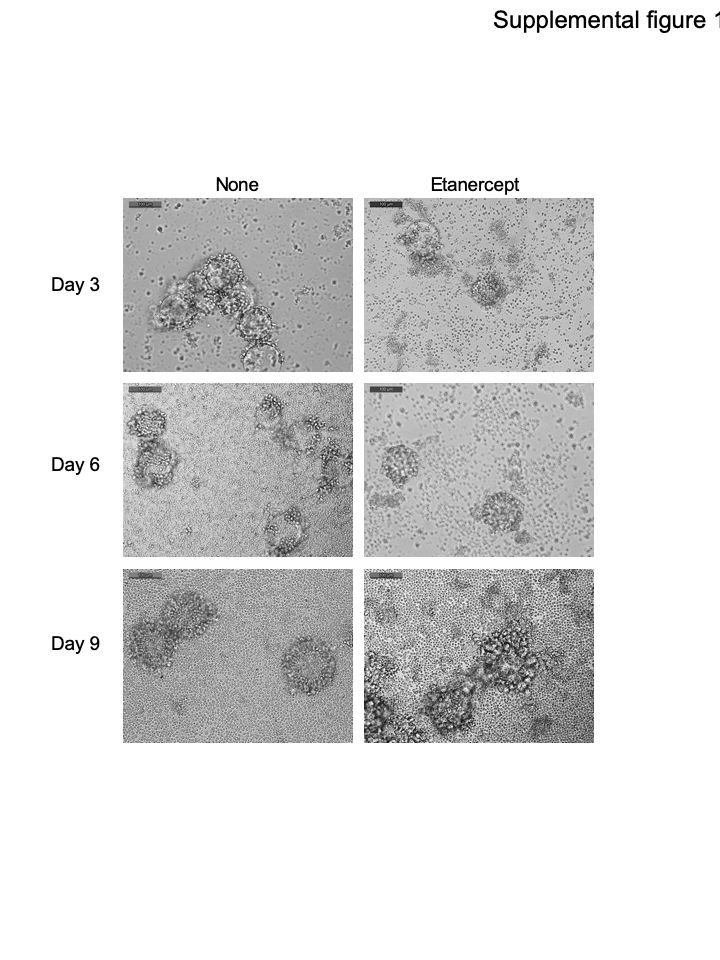

Supplement: Supplemental Figure 1 — The formation of tuberculous granuloma is not affected by etanercept. Isolated PBMCs from healthy donors were incubated with Sepharose beads coated with Mtb extracts for different periods of time in the presence or not of etanercept. Representative pictures of co-cultures at day 3, 6, and 9 are shown. [file Image_1.TIFF]
